# Lactylation-driven PDLIM1/PDAP1 axis remodels the inflammatory landscape of acute lung injury: mechanistic insights and precision intervention

**DOI:** 10.3389/fimmu.2026.1832309

**Published:** 2026-05-11

**Authors:** Hexiao Tang, Congkuan Song, Guiomar Correia, Changsheng Li, Daoquan Liu, Xuefeng Zhou

**Affiliations:** 1 Department of Thoracic Surgery, Zhongnan Hospital of Wuhan University, Wuhan, China; 2 Department of Cardiovascular and Thoracic Surgery, Cardinal Cardiopulmonary Diseases Hospital, Luanda, Angola

**Keywords:** acute lung injury, Ethyl methanesulfonate, lactylation, organoid, PDAP1, PDLIM1, progesterone

## Abstract

**Introduction:**

Acute lung injury (ALI) remains a lethal clinical challenge driven by an uncontrolled “cytokine storm” resulting from dysregulated inflammatory networks. The metabolic and molecular mechanisms orchestrating this process remain incompletely understood.

**Methods:**

We integrated multi-omics profiling with functional analyses in murine models and human iPSC-derived alveolar organoids. Key molecular players were identified through loss-of-function and pharmacological interventions, and the effects of a dual-target strategy using progesterone (PT) and ethyl methanesulfonate (EMS) were evaluated.

**Results:**

Metabolic reprogramming-driven lactylation emerged as a central orchestrator of inflammatory progression. PDAP1 lactylation acts as a pivotal metabolic switch for NLRP3 inflammasome activation and selective IL-1β release. Functional deficiency of PDLIM1 releases the molecular brake on NF-κB signaling, precipitating a broad-spectrum inflammatory cascade. These modifications bridge metabolic stress with oxidative damage via the NRF2/GPX4-mediated ferroptotic pathway. The “dual-target, dual-drug” intervention—PT targeting the PDLIM1 axis and EMS selectively disrupting PDAP1-mediated IL-1β maturation—effectively quelled systemic inflammation and attenuated ALI pathology in both in vivo and organoid models.

**Discussion:**

This study elucidates a novel metabolic-immune coupling mechanism in pulmonary polarization, shifting the focus from pan-inflammatory suppression toward precision immunomodulation. The findings provide a transformative theoretical paradigm for the management of ALI.

## Graphical Abstract

**Figure:**
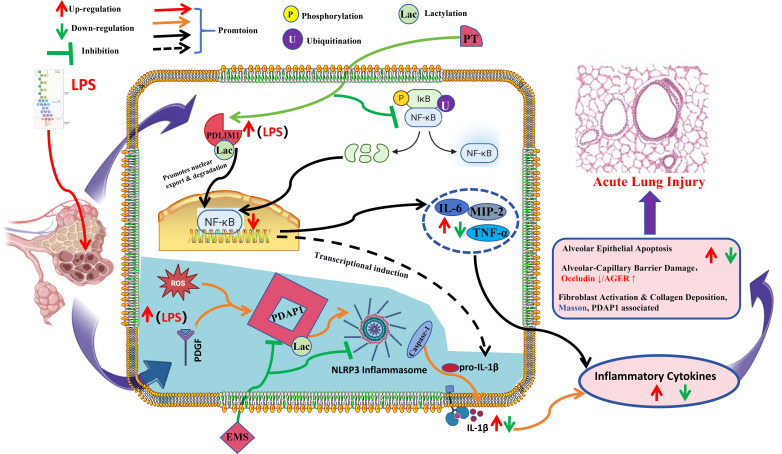

## 1 Introduction

Acute lung injury (ALI) and its clinical progression into Acute Respiratory Distress Syndrome (ARDS) represent a formidable global health challenge, defined pathologically by diffuse alveolar damage, microvascular hyperpermeability, and an escalating immunological firestorm (1, 2). Despite refinements in lung-protective ventilation and supportive care, the high mortality rate remains an intractable impasse, largely due to the cryptic regulatory circuits that transform localized inflammatory responses into systemic “cytokine storms” (2). Conventional therapeutic paradigms, which often rely on the blunt blockade of individual cytokines such as TNF-α or IL-6, frequently fail in clinical trials—likely because broad immunosuppression compromises essential host defense and impairs tissue-regenerative niches (3). Consequently, deciphering the molecular hierarchy within the inflammatory network to identify high-fidelity switches is imperative for transitioning toward precision immunomodulation in pulmonary critical care.

Immunometabolic rewiring is increasingly recognized as the primary driver of inflammatory polarization. In the injured pulmonary microenvironment, activated immune cells undergo a metabolic transition toward aerobic glycolysis, precipitating a surge in lactate levels (4, 5). Lactate has transcended its traditional identity as a metabolic byproduct to emerge as a potent signaling rheostat via lysine lactylation (Kla), which reshapes the transcriptional and functional landscape of the proteome (6). While histone lactylation has been implicated in macrophage polarization, the role of non-histone lactylation in systematically orchestrating the inflammatory signalscape—and whether specific lactylation-modified axes dictate the bimodal shift between localized and broad-spectrum inflammation—remains an uncharted frontier in immunobiology.

Grounded in the “metabolic reprogramming–lactylation–inflammatory network” nexus, this study integrates multi-omics profiling, lactylation proteomics, and computational pharmacology to dissect the stratified regulatory roles of Kla in ALI. PDZ and LIM domain 1 (PDLIM1) (7) and Platelet-Derived Growth Factor A associated protein 1 (PDAP1) (8) serve as pivotal molecular rheostats in the pulmonary inflammatory landscape, where PDLIM1 functions as a homeostatic scaffold that sequesters the NF-κB cascade, and PDAP1 acts as a metabolic-driven orchestrator of NLRP3 inflammasome activation and selective IL-1β secretion. We identify the PDLIM1/PDAP1 axis as a pivotal bimodal switch: lactylation of PDAP1 selectively drives NLRP3 inflammasome activation and IL-1β secretion, whereas the lactylation-dependent stabilization of PDLIM1 serves as a molecular brake on the NF-κB pathway, the loss of which triggers an unrestrained broad-spectrum cytokine storm. Guided by these insights, we propose a “dual-target, dual-drug” precision strategy, utilizing Ethyl methanesulfonate (EMS) and Progesterone (PT) to achieve multi-dimensional, graded modulation of the pulmonary inflammatory landscape. Validated in both murine models and high-fidelity human iPSC-derived lung organoids (9, 10), our work provides a mechanistic blueprint for transitioning from empirical immunosuppression to precision molecular phenotyping in ALI therapy.

## 2 Methods

### 2.1 Cross-platform multi-omics integration and lactylation regulatory landscape analysis

To decode the lactylation-modified landscape in ALI, we constructed a cross-scale bioinformatic integration framework.

Data integration and preprocessing: Single-cell RNA sequencing (scRNA-seq) datasets (GSE151263, GSE242127) and large-scale bulk transcriptomic data (GSE66890; ARDS n=29;Control n=28) were synergistically analyzed. A reference set of 336 lactylation-related genes was incorporated for targeted interrogation (11–13). All computational workflows were executed in R (v4.2.2).

Single-cell atlas construction: Data merging and batch-effect mitigation were performed using Seurat and Harmony, respectively. After rigorous quality control, 46,192 high-quality cells (genes<5000, mitochondrial genes<15%, ribosomal genes>3%, hemoglobin<0.1%) were retained. Following normalization, dimensionality reduction, and UMAP visualization, cell clusters were annotated using SingleR integrated with manual curation (resolution=0.8) to identify cluster-specific signatures (14, 15).

Lactylation scoring and functional correlation: Gene Set Variation Analysis (GSVA) was leveraged to calculate cell-specific lactylation scores. Compositional differences between high- and low-score groups were analyzed, and correlations with Hallmark pathways were interrogated via GSVA (16).

Differential expression and enrichment analysis: Bulk transcriptomic analysis (limma) identified 748 upregulated and 625 downregulated genes (P<0.05, |logFC|>0.1), while scRNA-seq yielded 4,007 upregulated and 408 downregulated genes. The intersection of these sets with the lactylation reference genes identified 14 differentially expressed lactylation-related genes, which were subjected to GO and KEGG enrichment analysis.

**Figure 1:**
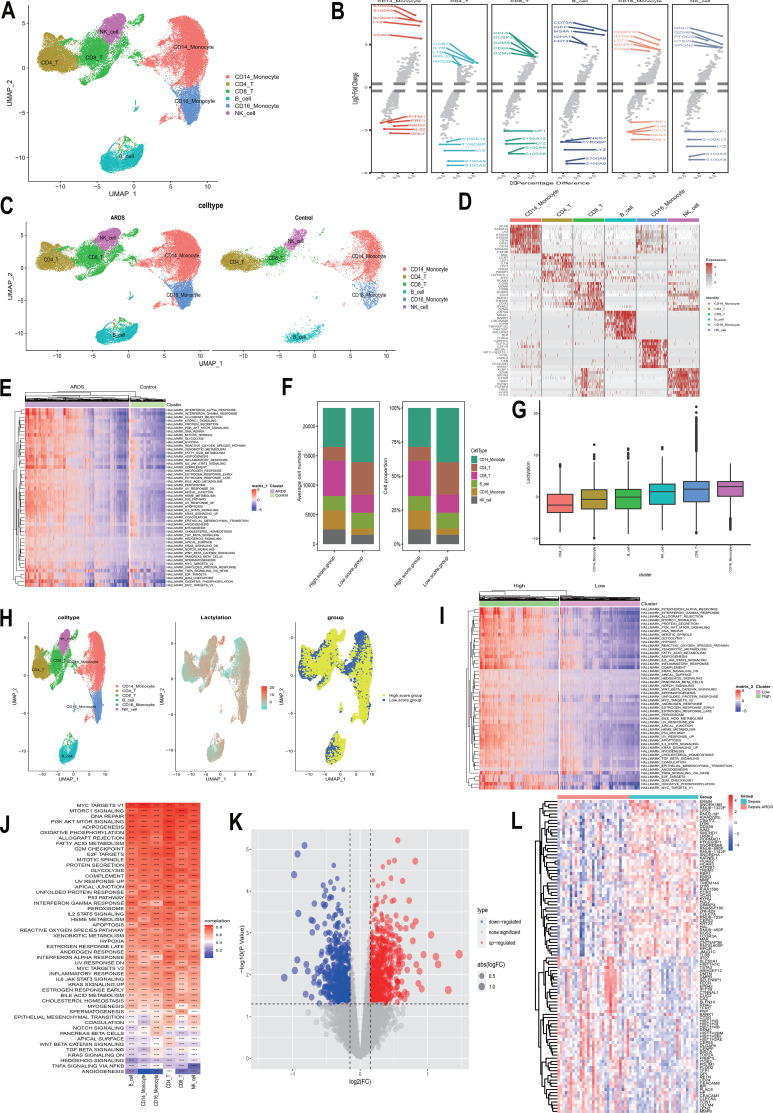
Integrative scRNA-seq and bulk transcriptomic analysis decodes the lactylation-associated landscape of ALI. **(A)** UMAP visualization and cell-type annotation of the single-cell atlas. **(B)** Top 5 differentially expressed markers (up- and down-regulated) across cell clusters. **(C)** UMAP projection illustrating cell distribution in Control versus ARDS groups. **(D)** Heatmap highlighting the top 10 marker genes for each cell type identified via the COSG algorithm. **(E)** GSVA-derived heatmap of Hallmark pathway activities between experimental groups. **(F, G)** Distribution of lactylation scores across cell types **(F)** and quantitative analysis of cell counts and proportions in high- versus low-lactylation score groups **(G)**. **(H)** Spatial expression patterns of lactylation scores projected onto UMAP (High vs. Low groups). **(I)** Heatmap of GSVA scores for Hallmark pathways stratified by lactylation activity. **(J)** Correlation heatmap between lactylation scores and Hallmark signaling cascades. **(K, L)** Differential expression analysis using the GSE66890 cohort: Volcano plot highlighting up- (red) and down-regulated (blue) genes **(K)**; Heatmap illustrating the transcriptional landscape of sepsis-associated ARDS versus sepsis controls **(L)**.

**Figure 2:**
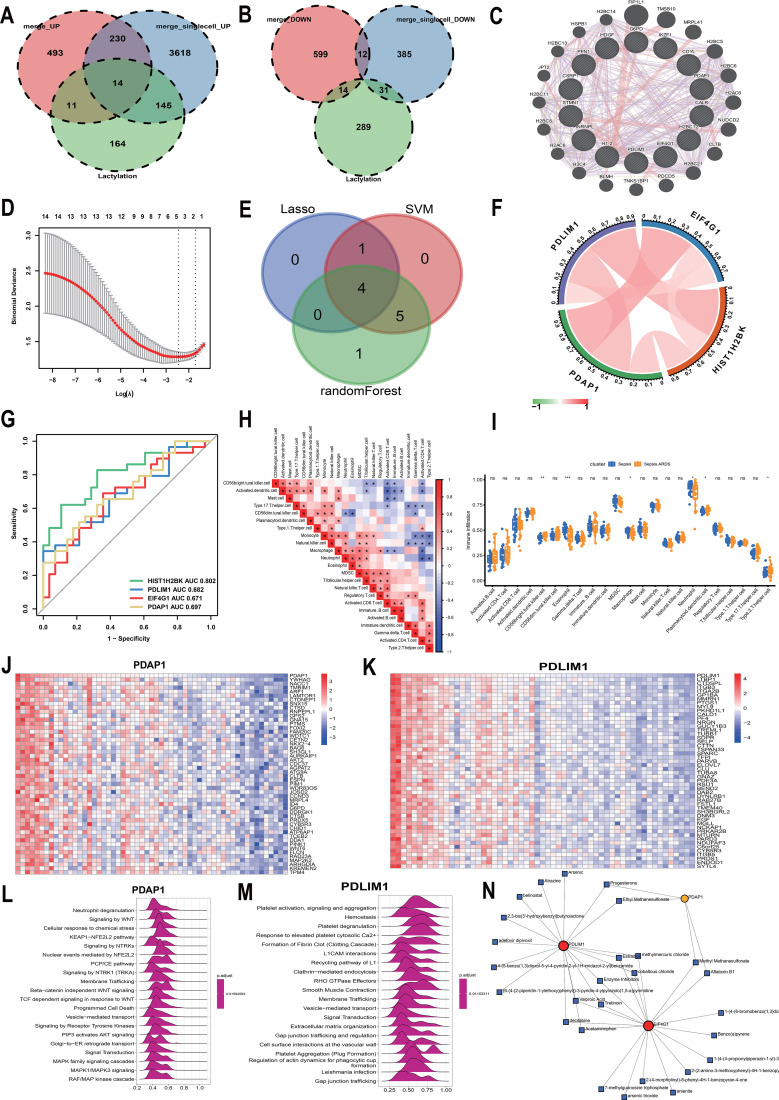
Computational identification, diagnostic validation, and functional mapping of core lactylation-related hubs. Single-cell differential analysis identified 4,007 up-regulated and 408 down-regulated genes (P. Val < 0.05, |logFC| > 0.15). **(A, B)** Venn diagrams showing the intersection of differentially expressed genes and the lactylation gene set: 14 up-regulated genes crossed over **(A)**, while no intersection was found for down-regulated genes **(B)**. **(C)** Protein-protein interaction (PPI) network of the 14 candidates constructed via GENEMANIA for machine learning prioritization. **(D, E)** Multi-algorithmic feature selection: LASSO regression initially narrowed the set to 5 genes **(D)**, with subsequent ensemble intersection yielding 4 core hubs **(E)**. **(F)** Heatmap showing correlations among the 4 core genes (red: positive, green: negative). **(G)** ROC curves evaluating the diagnostic fidelity of core genes via the pROC package. **(H, I)** Characterization of the immune infiltration landscape via ssGSEA: Correlation matrix of immune cell subsets **(H)** and differential infiltration analysis between sepsis-induced ARDS and sepsis controls **(I)** (ns p>0.05, *p<0.05, **p<0.01, *p<0.001). **(J, K)** Genome-wide correlation analysis for PDAP1 and PDLIM1, with heatmaps displaying the top 50 positively correlated genes. **(L, M)** Single-gene GSEA (Reactome database, Top 20 pathways) for PDAP1 and PDLIM1; positive/negative enrichment scores indicate respective pathway correlations. **(N)** Prediction of potential therapeutic compounds targeting the 4 core hubs via the NetworkAnalyst database.

Core target identification and validation: A protein-protein interaction (PPI) network was constructed based on the 14 candidate genes. Core genes were prioritized using an ensemble of machine learning algorithms, including LASSO, Random Forest, and Support Vector Machine (SVM). Diagnostic efficacy was evaluated via ROC curves, and immune infiltration landscapes were characterized via ssGSEA. Upstream regulatory networks (miRNAs, transcription factors) and potential therapeutic compounds were predicted using NetworkAnalyst. Based on integrated genomic signatures, pharmacological screening, and clinical relevance (**Figures 1**, **2**; **Supplementary Figures S1**-**S3**), drug screening results, and clinical application information, we finalized our focus on PDAP1 and PDLIM1 as core targets, and EMS and PT as lead intervention candidates.

### 2.2 Murine ALI modeling and precision intervention

ALI was induced in mice as previously described (17, 18). SPF-grade male BalB/C mice (6 weeks old) were housed in a standardized environment (20–26 °C, 30–70% humidity, 12h light/dark cycle). Following a 7-day acclimatization, mice were randomized into four groups (n=5): Control (intratracheal PBS), LPS (LPS, intratracheal, 5 mg/kg), LPS+PT, and LPS+EMS. PT (1 mg/mL in corn oil) and EMS (4 mg/mL in saline) were administered via intraperitoneal injection (10 μL/g) every 48 hours for three doses (19–21). For cell-type-specific genetic interrogation, AAV6 vectors carrying shRNA-PDLIM1 or shRNA-PDAP1 under the SPC promoter were utilized to target alveolar type II epithelial cells (22, 23). AAV6 was delivered intranasally (1×10¹¹ vg/mL) 14 days prior to LPS challenge. On day 7 post-LPS, bronchoalveolar lavage fluid (BALF) and lung tissues were harvested. Left lungs were fixed in 4% PFA for histomorphometry, while right lungs were flash-frozen at -80 °C for molecular analysis. All procedures were approved by the Institutional Animal Care.

### 2.3 Human iPSC-derived alveolar organoid modeling and pharmacological rescue

Organoid Differentiation: Human iPSCs were differentiated into alveolar-like organoids (ALOs) following established protocols (9, 10). Upon maturation, ALOs were stratified into four groups: Control, LPS (100 ng/mL), LPS+EMS (36 μg/mL), and LPS+PT (9 μg/mL). Control ALOs were maintained in alveolar maturation medium. For injury groups, LPS was administered for 7 days with media changes every 3 days. Rescue groups (EMS and PT) received pharmacological treatment for 7 days following the initial LPS challenge to evaluate therapeutic efficacy in a human context. Samples were harvested for Western blotting and immunofluorescence (IF).

### 2.4 Histopathological characterization and semi-quantitative scoring

Lung sections were processed for H&E and Masson’s trichrome staining to evaluate inflammatory infiltration and collagen deposition, respectively (24). Barrier integrity and epithelial markers were interrogated via IF (25) using antibodies against AGER (Proteintech, 83759-4-RR, 1:500), Occuludin (Proteintech, 80545-1-RR, 1:500), and SP-B (Proteintech, 82866-16-RR, 1:200). A double-blind scoring system was employed based on staining intensity (1-4) and percentage of positive cells (1-4). Results were categorized by the product of these scores: negative (≤4), weak positive (4–8), moderate positive (8–12), and strong positive (12–16) (25–27).

### 2.5 Western blotting and signal transduction analysis

Total protein was extracted from lung tissues and organoids to assess signal transduction cascades and metabolic markers (28). Primary antibodies included GPX4 (Proteintech, 67763-1-lg, 1:2000), ACSL4 (Proteintech, 22401-1-AP, 1:20000), NRF2 (Proteintech, 16396-1-AP, 1:2000), KEAP1 (Proteintech, 10503-2-AP, 1:5000), PDLIM1 (Proteintech, 11674-1-AP, 1:3000), PDAP1 (Proteintech, 15081-1-AP, 1:800), P65 (Proteintech, 10745-1-AP, 1:3000), p-P65 (Proteintech, 82335-1-RR, 1:5000), and H3K18la (PTMab, PTM-1427RM, 1:800). GAPDH served as the loading control.

### 2.6 Inflammatory landscape quantification (ELISA & qPCR)

Cytokine concentrations (IL-1β, IL-6, TNF-α, MIP-2) in BALF and organoid supernatants were quantified via ELISA (29) (Nanjing BYabscience technology Co.,Ltd, BY-EM220174/BY-EM220188/BY-EM220852/BY-EM220428). Transcriptional signatures were interrogated via RT-qPCR following total RNA extraction and reverse transcription (24, 29). Primer sequences: IL-1β (F: 5’-GTAATGAAAGACGGCACACCC, R: CAGGCTTGTGCTCTGCTTGTG-3’); IL-6 (F: 5’-CATAGCTACCTGGAGTACATGAAGAA, R: GACTCCAGCTTATCTCTTGGTTGA-3’); TNF-α (F: 5’-CCCTCACACTCACAAACCACC, R: CTTTGAGATCCATGCCGTTG-3’); GAPDH (F: 5’-CCTCGTCCCGTAGACAAAATG, R: TGAGGTCAATGAAGGGGTCGT-3’).

### 2.7 Statistical analysis

Data are presented as Mean ± SD. Statistical inferences were performed using IBM SPSS and visualized via GraphPad Prism 9.0 (25). Inter-group differences were evaluated using one-way ANOVA followed by *post-hoc* tests, while two-group comparisons were analyzed via unpaired two-tailed Student’s t-tests. P<0.05 was considered statistically significant.

## 3 Results

### 3.1 Multi-omics integration decodes the single-cell lactylation landscape and identifies core regulatory hubs

To systematically resolve the immunometabolic remodeling during ALI progression, we integrated scRNA-seq, bulk transcriptomics, and a specialized lactylation-modified gene set to construct a comprehensive intrapulmonary lactylation regulatory atlas (**Figure 1**; **Supplementary Figure S1**). Utilizing the Harmony algorithm to mitigate batch effects, we identified a high-resolution landscape of immune cell heterogeneity (**Figures 1A–D**). GSVA analysis revealed that lactylation scores exhibit significant spatiotemporal dynamics within the immune cell populations of ALI patients, closely coupling with glycolysis, angiogenesis, and multiple inflammatory signaling cascades (**Figures 1E–J**). Through the intersection of the ALI clinical cohort and single-cell differentially expressed genes, we narrowed down 14 key lactylation-associated candidates (**Figures 2A–C**). We subsequently employed an ensemble of machine learning algorithms—including LASSO regression, Random Forest (RF), and Support Vector Machine (SVM)—for multi-dimensional feature reduction, ultimately defining PDLIM1, PDAP1, HIST1H2BK, and EIF4G1 as the core characteristic genes driving ALI evolution (**Figures 2D–F**). ROC curve analysis confirmed the high diagnostic fidelity of this gene set (**Figure 2G**), and their expression levels correlated significantly with inflammatory infiltrates, such as neutrophils and monocytes (**Figures 2H, I**). Further computational docking and pharmacological sensitivity profiling identified EMS and PT as agents with precision targeting potential for the PDLIM1 and PDAP1 axes, respectively (**Figure 2N**; **Supplementary Figures S2**, **S3**), providing a molecular foundation for “metabolic-epigenetic” intervention. Analyses of HIST1H2BK and EIF4G1 are detailed in **Supplementary Figures S2**, **S3**. Based on these genomic signatures and clinical relevance, we finalized the PDLIM1 and PDAP1 axes and their respective lead compounds for downstream mechanistic interrogation.

### 3.2 LPS-driven metabolic-epigenetic cascades trigger PDLIM1/PDAP1 axis dysregulation and oxidative network collapse

**Figure 3:**
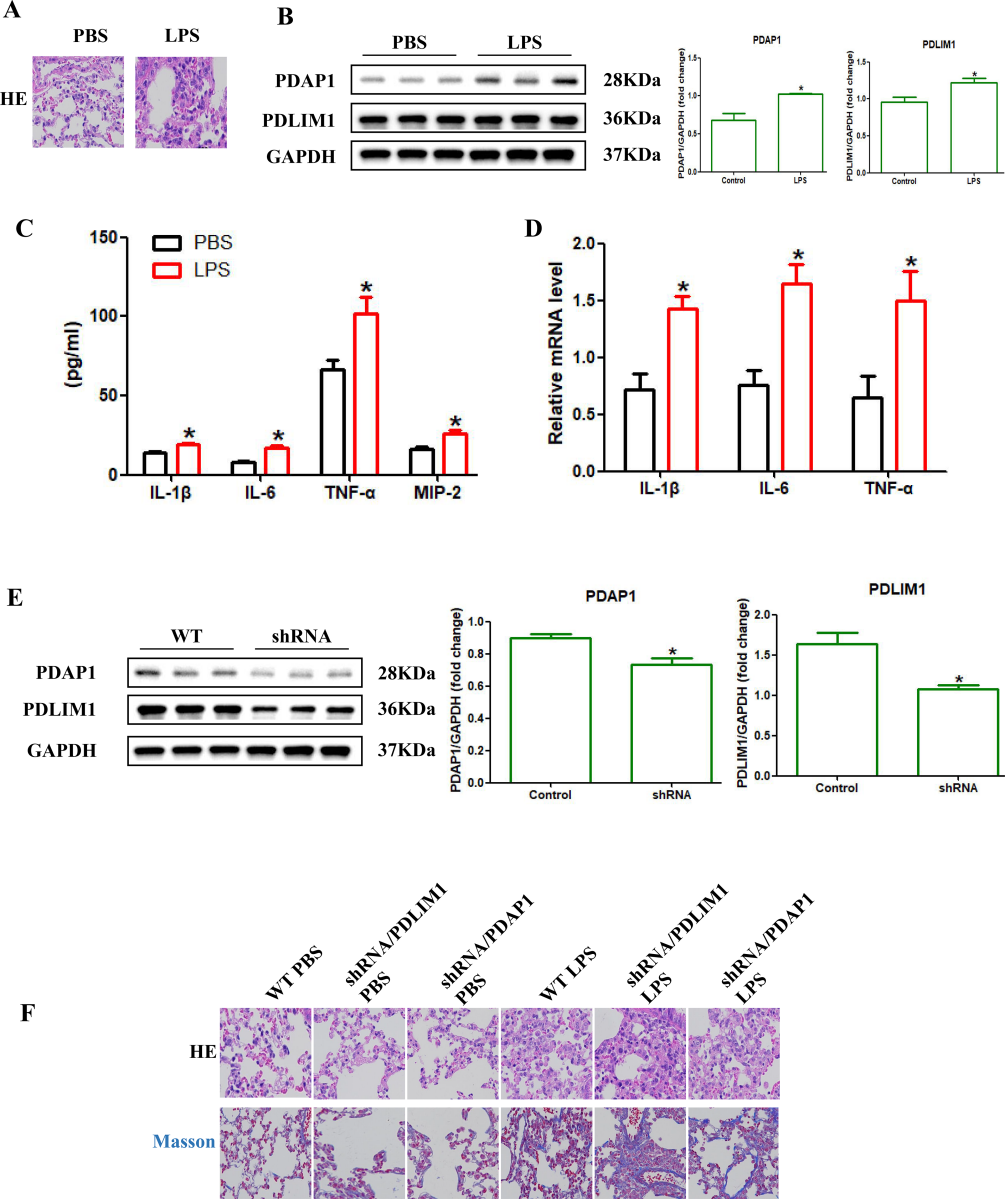
Pathological expression of PDLIM1/PDAP1 and their impact on the inflammatory phenotype in murine ALI. **(A, B)** Western blot quantification confirms significant upregulation of PDLIM1 and PDAP1 in the LPS-induced murine lung injury model versus controls (*p<0.05). **(C, D)** Elevated levels of inflammatory cytokines in bronchoalveolar lavage fluid (BALF) **(C)** and lung tissue **(D)** following LPS challenge (*p<0.05). **(E)** Validation of alveolar type II (ATII) epithelial-specific knockdown of PDLIM1 and PDAP1 using AAV6 vectors under the SPC promoter (*p<0.05). **(F)** Representative H&E and Masson’s trichrome staining (blue: collagen deposition) illustrating the lung injury and pro-fibrotic patterns in the LPS model.

**Figure 4:**
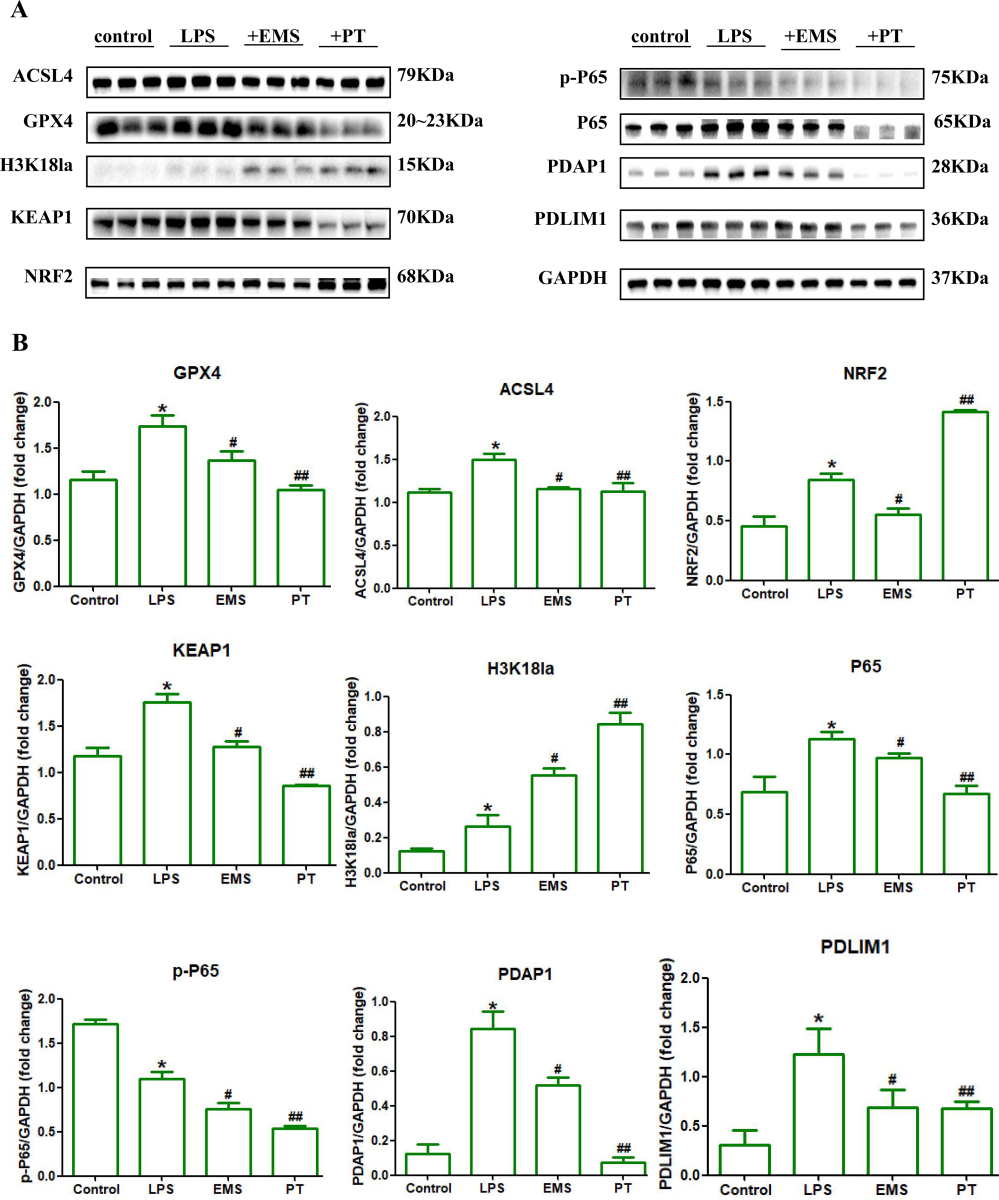
Mechanistic validation of the lactylation-driven PDLIM1/PDAP1 axis as a molecular rheostat in human ALOs. **(A, B)**. Western blot analysis **(A)** and semi-quantitative profiling **(B)** of key signaling molecules within the PDLIM1/PDAP1 axis following intervention with EMS and PT. These data confirm the axis serves as a critical regulator of inflammatory signaling and proteostasis in humanized pulmonary environments (*p<0.05, #p<0.05, ##p<0.05).

In the LPS-induced murine ALI model, we observed a profound remodeling of the pulmonary metabolic-epigenetic landscape. Western Blotting analysis revealed that LPS challenge significantly upregulated the histone lactylation marker H3K18la, signaling a global enhancement of lactylation modifications precipitated by lactate accumulation in the pulmonary microenvironment (**Figures 3A, B**, **4A, B**). Amidst this lactylation surge, the core regulatory hubs PDAP1 and PDLIM1 both exhibited pathological overexpression (**Figures 3A, B**), accompanied by a total increase in the NF-κB subunit P65 and a dynamic shift in its phosphorylation (p-P65) levels (**Figures 4A, B**). Mechanistically, we propose a biochemical hierarchy where PDLIM1 acts as a molecular scaffold sequestering p65 in the cytoplasm. Its downregulation releases this ‘molecular brake,’ triggering p-P65-mediated transcription of pro-inflammatory factors and ACSL4. This inflammatory surge, coupled with lactylation-induced metabolic shifts, overwhelms the NRF2/GPX4 antioxidant system, leading to the collapse of cellular redox homeostasis. Thus, PDLIM1 serves as a critical upstream rheostat whose loss precipitates a cascade of oxidative network failure and subsequent ferroptosis.

**Figure 5:**
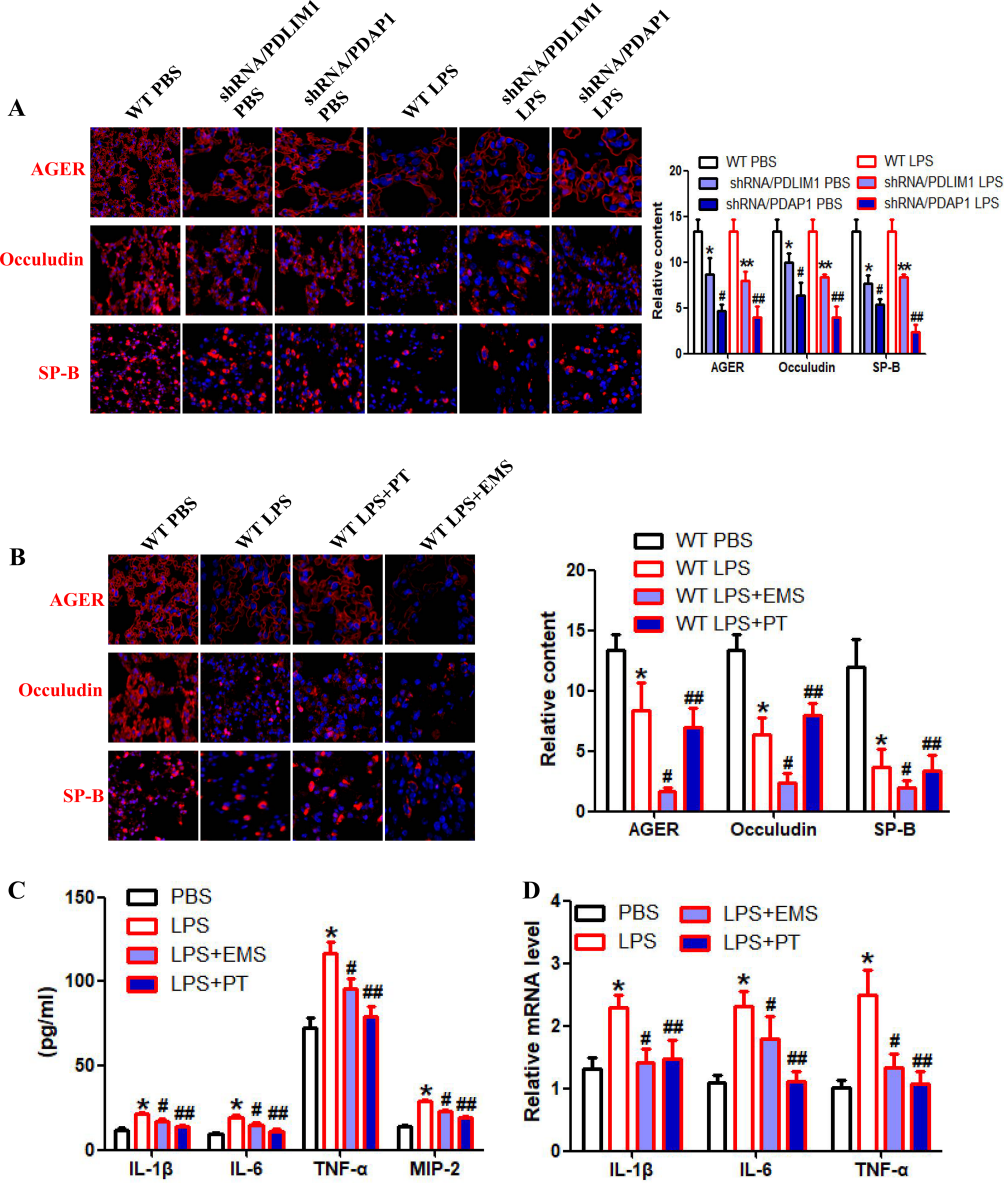
Precision intervention with EMS and PT quells the inflammatory cascade and restores barrier integrity. **(A, B)** Lung-specific silencing of PDLIM1 or PDAP1 exacerbates the loss of AGER, Occludin, and SP-B, while treatment with EMS or PT significantly restores these markers in LPS-challenged mice (*p<0.05, ∗∗p<0.05, #p<0.05, ##p<0.05). **(C, D)** Graded reduction of inflammatory cytokines in BALF **(C)** and lung tissue **(D)** following EMS and PT intervention, consistent with the observed proteomic trends (*p<0.05, #p<0.05, ##p<0.05).

Concurrent monitoring revealed that lactylation imbalance triggered a complex collapse of cellular proteostasis: the pro-ferroptotic protein ACSL4 was markedly elevated, while key antioxidant defense components, including GPX4, NRF2, and the oxidative stress sensor KEAP1, showed compensatory upregulation (**Figures 4A, B**). This suggests that the pulmonary tissue attempts to initiate an antioxidant protective program under lactylation pressure, which nonetheless fails to offset the overwhelming inflammatory damage. Macroscopically, this molecular disarray resulted in a comprehensive “cytokine storm” in the bronchoalveolar lavage fluid (BALF), characterized by the surge of IL-1β, IL-6, TNF-α, and MIP-2 (**Figures 3C, D**). IF confirmed the breakdown of alveolar integrity, evidenced by high AGER expression, Occuludin fragmentation, and SP-B depletion (**Figures 5A, B**). Causality was further established via AAV-mediated lung-specific silencing (shRNA-PDLIM1/shRNA-PDAP1), demonstrating that dysregulation of this dual axis is a central mechanism inducing the composite injury phenotype and pro-fibrotic tendency in ALI (**Figures 3E, F**, **5A**).

### 3.3 Pharmacological reprogramming of the lactylation landscape facilitates precise inflammatory network “reset”

To explore precision intervention pathways, we evaluated the remodeling effects of EMS and PT on the lactylation-inflammatory network. Our experiments yielded a striking observation: PT intervention significantly further induced super-physiological levels of H3K18la; remarkably, this “reinforced lactylation” effect correlated positively with the broad-spectrum suppression of inflammation (**Figures 4A, B**, **5C, D**). Results showed that PT not only significantly downregulated the expression of PDAP1, PDLIM1, and P65/p-P65 (**Figures 4A, B**) but also effectively halted the release of the entire inflammatory cytokine repertoire (IL-1β, IL-6, TNF-α, MIP-2) in the BALF (**Figures 5C, D**). To distinguish lactate-driven effects from direct TLR4-mediated transcriptional repression, we observed that enhancing H3K18la to super-physiological levels via Progesterone (PT) treatment effectively reset the PDLIM1/PDAP1 axis and quelled inflammation. This suggests that the axis is intrinsically sensitive to the homeostatic logic of the lactylation landscape rather than being a linear downstream slave to LPS-NF-κB signaling. This metabolic feedback loop provides a secondary layer of gene regulation that can override or fine-tune primary inflammatory signals initiated by LPS.

In contrast, EMS exhibited a distinct “selective” regulatory profile: it precisely targeted and reduced PDAP1 protein abundance, primarily interfering with the specific injury cascade mediated by IL-1β (**Figures 4A, B**, **5C, D**). Regarding cytoprotection, PT intervention significantly induced a peak in NRF2 expression and synergistically downregulated ACSL4 and KEAP1, effectively repairing the ferroptotic and oxidative damage triggered by the initial lactylation imbalance. Histopathological evaluations (H&E and Masson’s staining, **Figure 3F**) consistently confirmed that both agents significantly alleviated pulmonary edema, inflammatory infiltration, and early collagen deposition. These findings demonstrate that targeting different nodes of the lactylation-driven dual axis allows for precision control of the ALI inflammatory network, ranging from “broad-spectrum quelling” to “selective attenuation.” By directly depleting microenvironmental lactate, such enzymatic strategies could theoretically prevent the initiation of the H3K18la-PDLIM1/PDAP1 cascade (30–32). Incorporating these lactate-scavenging approaches would provide a direct ‘metabolic clearance’ route to restore the pulmonary inflammatory landscape, offering a synergistic complement to the pharmacological interventions (PT and EMS) explored in this study.

### 3.4 Human iPSC-derived alveolar organoids confirm the evolutionary conservation and translational potential of the lactylation-driven axis

**Figure 6:**
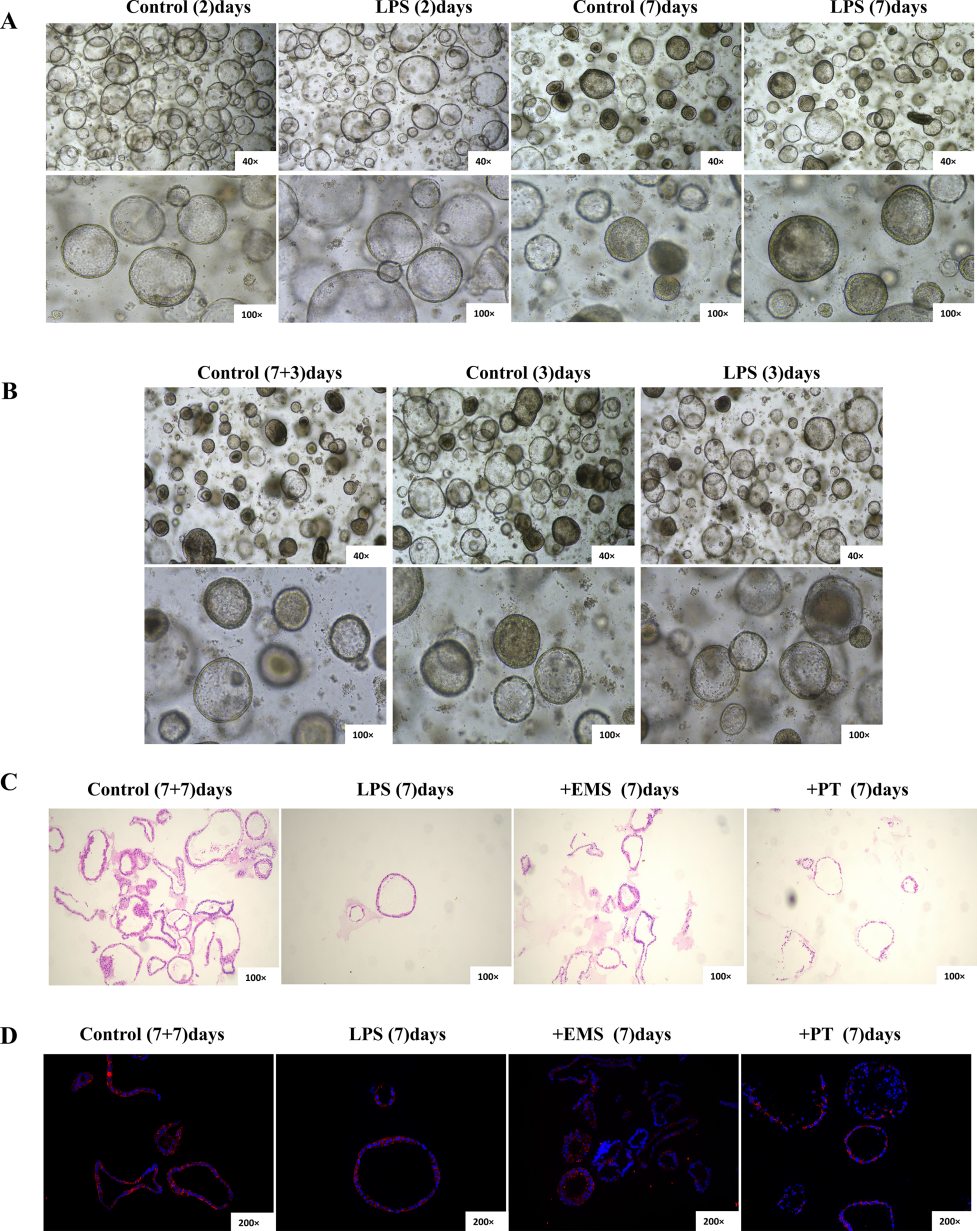
Recapitulation of the ALI injury landscape and pharmacological rescue in human iPSC-derived alveolar organoids (ALOs). **(A, B)** Step-wise induction of iPSCs into ALOs and establishment of the humanized injury model via LPS stimulation. **(C, D)** Evaluation of therapeutic efficacy of EMS and PT through H&E staining **(C)** and immunofluorescence microscopy **(D)**, confirming morphological rescue and epithelial marker restoration in human alveolar niches.

To bridge the species gap between animal models and clinical application, we validated the universality of this mechanism in high-fidelity human iPSC-derived ALOs (**Figure 6**). Within the LPS-induced humanized injury landscape, ALOs perfectly recapitulated the pathological progression of H3K18la elevation, PDLIM1/PDAP1 overexpression, and p-P65 signaling activation (**Figures 4A, B**). Critical intervention experiments demonstrated that EMS and PT synergistically restored proteostatic balance within the PDLIM1/PDAP1 axis, significantly inhibiting NF-κB transcriptional activity and effectively recovering the spatial distribution of marker proteins in the damaged epithelium (**Figures 4B**, **6C, D**). This consistency between murine models and human organoids confirms that the lactylation-driven PDLIM1/PDAP1 axis is a highly conserved “molecular rheostat” in ALI evolution. Our study not only elucidates a novel metabolic-epigenetic coupling mechanism regulating inflammation but also provides a robust evidence chain for developing precision anti-inflammatory strategies based on lactylation molecular phenotyping.

## 4 Discussion

This study deciphers the molecular logic by which Kla orchestrates the evolution of inflammatory networks in ALI. By integrating cross-scale omics with functional validation, we identified the PDLIM1/PDAP1 axis as a central metabolic-epigenetic rheostat. Our findings transcend the traditional view of pathway-specific inhibition, proposing a novel theoretical paradigm of “Inflammatory Network Reprogramming” achieved through metabolic rewiring. While protein lactylation is a global metabolic signature under inflammatory stress, our study specifically prioritized the PDLIM1/PDAP1 axis using an ensemble of three machine learning algorithms (LASSO, Random Forest, and SVM). This rigorous filtering process ensured that among numerous lactylated candidates, PDLIM1 and PDAP1 emerged as the most robust diagnostic hubs with the highest correlation to immune cell infiltration. Thus, rather than being a non-specific byproduct of metabolic flux, this axis represents a high-leverage ‘molecular bottleneck’ that uniquely orchestrates the magnitude of the inflammatory response in ALI.

Since the landmark discovery of Kla as a bridge between glycolysis and gene transcription (33), research has primarily focused on its “timer” effect in resolving late-stage inflammation, such as driving M2 macrophage polarization (34). However, we have significantly expanded this cognitive boundary. Our data demonstrate that in the hyper-acute phase of ALI, the pathological surge of H3K18la serves not merely as a marker of metabolic stress but as an epigenetic harbinger that drives the ensuing cytokine storm. Crucially, we show that the regulatory reach of Kla extends beyond the classical histone landscape to non-histone signaling hubs, specifically PDLIM1 and PDAP1. This reinforces the emerging concept of lactylation as a ubiquitous functional rheostat (35). This hierarchical control—spanning from the transcriptional landscape to protein stability—explains how lactate accumulation acts as a “metabolic instruction” to trigger complex intrapulmonary immunological cascades (**Graphical Abstract**). A critical consideration arising from our findings is the precise temporal and mechanistic hierarchy between metabolic reprogramming and the observed dysregulation of the PDLIM1/PDAP1 axis. Our data suggest that the elevation of H3K18la is not merely a concomitant marker of cellular stress, but rather a primary epigenetic ‘harbinger’ that dictates the transcriptional fate of inflammatory hubs. Under the acute metabolic shift toward aerobic glycolysis—characteristic of the early phase of ALI—lactate accumulation serves as the requisite substrate for histone lactylation. This modification potentially reshapes the chromatin accessibility at the promoter regions of PDLIM1 and PDAP1, or directly modulates their protein stability, thereby acting as a metabolic-epigenetic rheostat. Consequently, the lactylation-driven remodeling of this axis precedes the full-scale commitment to a ‘cytokine storm,’ positioning metabolic intervention as a high-leverage strategy to intercept the inflammatory cascade before it reaches a point of irreversibility.

The convergence of NF-κB-driven transcriptional bursts and oxidative stress-induced cell death represents the two pillars of ALI pathology (36). The PDLIM1/PDAP1 dual-axis model established here provides a high-fidelity molecular explanation for the integration of these pathways. PDLIM1, acting as a molecular brake on the NF-κB pathway, mirrors its role previously identified in autoimmunity and malignancy (7). Our Western blot (WB) data reveal a profound biological insight: under LPS challenge, the surge in Kla is accompanied by a compensatory upregulation of antioxidant defense proteins, including NRF2, GPX4, and KEAP1 (**Figure 4**). While this “defensive surge” reflects the tissue’s innate attempt at self-preservation, it remains insufficient to arrest the p-P65-mediated inflammatory polarization. We propose that PDAP1 lactylation serves as a “feed-forward amplifier” for the NLRP3 inflammasome, while PDLIM1 exhibits functional heterogeneity. The resulting bimodal imbalance triggers the elevation of the pro-ferroptotic core protein ACSL4, suggesting that Kla couples inflammatory signaling directly to ferroptosis and antioxidant failure. This integrated landscape provides a mechanistic basis for the clinical paradox of concurrent inflammatory flare-ups and impaired tissue repair in ALI patients. In this study, we employed both murine models and human alveolar-like organoids (ALOs) to validate the PDLIM1/PDAP1 axis. While murine models effectively capture the systemic complexity of ALI—including the recruitment of circulating neutrophils and the progression of fibroproliferation—human ALOs provide a refined platform to observe cell-intrinsic metabolic-epigenetic responses within a humanized genetic context. The consistency observed across these systems suggests that the lactylation-driven remodeling of the inflammatory landscape is an evolutionarily conserved mechanism. By integrating the systemic physiological insights from mice with the high-fidelity molecular data from human organoids, we provide a more robust translational framework for evaluating potential therapeutic interventions in acute lung injury.

The recurring failure of empirical immunosuppression in ALI/ARDS trials stems from the blunt inhibition of single targets, which often compromises essential immune surveillance (37). Our proposed “Metabolic Feedback-Based Precision Reprogramming” offers a transformative alternative. Remarkably, PT intervention did not merely suppress lactylation; instead, it induced “super-physiological” levels of H3K18la. This aligns with its known anti-inflammatory profile but anchors the effect to a specific metabolic-immune target for the first time (7). In this context of reinforced lactylation, PT effectively reset the pathological levels of PDAP1, PDLIM1, and p-P65 while driving NRF2 expression to its peak, synergistically inhibiting ACSL4. This suggests that PT leverages the “homeostatic feedback” logic of lactylation—accelerating lactylation-driven gene programs to prematurely terminate the inflammatory storm and reboot antioxidant defenses. In contrast, the selective inhibition of the PDAP1 axis by EMS demonstrates the “tailorability” of the inflammatory network. This “dual-target, dual-drug” success heralds a transition from non-specific immunosuppression to “graded intervention” based on metabolic phenotyping, potentially utilizing BALF lactylation levels or PDLIM1/PDAP1 signatures to guide clinical stratification. We have refined the concept of ‘precision intervention’ to reflect a tailored therapeutic framework. Unlike classical models where histone lactylation serves as a late-stage ‘timer’ for inflammation resolution, our findings reveal its role as a hyper-acute driver of the cytokine storm. By identifying this metabolic-epigenetic switch, we provide a basis for stratified therapy: broad-spectrum suppression via Progesterone-induced homeostatic resetting, or targeted NLRP3 attenuation via EMS. This distinction positions our axis as a novel conceptual expansion of lactylation’s role in acute pulmonary pathology.

Despite the mechanistic insights provided, several limitations warrant acknowledgment. First, while our study highlights the global surge in lactylation, future research utilizing spatial transcriptomics and single-cell proteomics is required to map the precise spatiotemporal distribution of the PDLIM1/PDAP1 axis across distinct pulmonary niches. Second, the specific ‘writers’ and ‘erasers’—the enzymatic machinery responsible for the addition and removal of lactyl groups on these core hubs—remain to be fully characterized in the context of human ALI. Furthermore, while the Human Alveolar Like Organoids (ALOs) offer a robust humanized platform, they do not fully encapsulate the systemic complexity of inter-organ crosstalk during sepsis. Addressing these gaps will be essential for refining the precision of metabolic-epigenetic interventions and translating these findings into clinical therapies for critically ill patients. In summary, this study elevates the mechanistic understanding of ALI from a linear pathway perspective to a complex network regulation model, identifying the lactylation-driven PDLIM1/PDAP1 axis as a definitive molecular rheostat. Our work provides a new framework for deciphering the heterogeneity of lung injury and establishes a foundational theory for precision immunotherapy through metabolic-epigenetic reprogramming.

## Data Availability

The datasets presented in this study can be found in online repositories. The names of the repository/repositories and accession number(s) can be found in the article/**Supplementary Material**.

## Supplementary material

Supplementary Figure 1 Single-cell data preprocessing and rigorous quality control. scRNA-seq data were integrated from GEO (GSE151263 and GSE242127) and bulk data from GSE66890. The 336 lactylation genes were curated from high-impact literature (PMIDs: 37242427, 35761067, 36092712). Analysis used R 4.2.2 and the Seurat package with Harmony batch-effect mitigation. Quality control (QC) criteria: the number of genes detected per cell (nFeature-RNA) is less than 5000, the mitochondrial gene expression ratio (percent_ito) is less than 15%, the ribosomal gene expression ratio (percent_ribo) is greater than 3%, and the hemoglobin gene expression ratio (percent_rb) is less than 0.1%. 46,192 cells were retained. **(A, B)** Violin plots of nFeature_RNA and nCount_RNAn pre- and post-QC. **(C, D)** Distribution of mitochondrial, ribosomal, and hemoglobin genes pre- and post-QC. **(E)** Correlation between nFeature_RNA and nCount_RNA, indicating high data fidelity. Cell types were annotated using SingleR and manual curation (Resolution 0.8).

Supplementary Figure 2 Systematic bioinformatics pipeline for core gene prioritization. **(A)** Cellular composition per sample. **(B, C)** Proportional distribution and trends of cell types across groups. **(D, E)** KEGG and Reactome pathway enrichment of top 100 cluster markers. **(F)** GO (BP, CC, MF) and KEGG enrichment of differentially expressed genes (DEGs). **(G)** Summary of single-cell differential analysis (4,007 up-regulated, 408 down-regulated DEGs). **(H, I)** Functional GO and KEGG enrichment of the intersected DEGs. **(J, K)** Top 10 genes identified via SVM **(J)** and Random Forest importance ranking **(K)**. **(L)** Correlation map between the 4 core hubs and significantly infiltrating immune cells (ssGSEA; dot size: coefficient; color: pp-value).

Supplementary Figure 3 Upstream regulatory networks and functional enrichment of EIF4G1 and HIST1H2BK. **(A, B)** Genome-wide correlation heatmaps for EIF4G1 and HIST1H2BK, highlighting the top 50 co-expressed genes. **(C, D)** Single-gene Reactome GSEA (Top 20 pathways) for EIF4G1 and HIST1H2BK; enrichment scores indicate respective pathway correlations. **(E, F)** Upstream miRNA-TF regulatory networks for the core hubs predicted via NetworkAnalyst (displaying significant nodes).
