# Supplementary figures and images for: Lactylation-driven PDLIM1/PDAP1 axis remodels the inflammatory landscape of acute lung injury: mechanistic insights and precision intervention
Source: Front Immunol. 2026 May 11;17:1832309. doi: 10.3389/fimmu.2026.1832309 (PMC13199081; doi:10.3389/fimmu.2026.1832309)

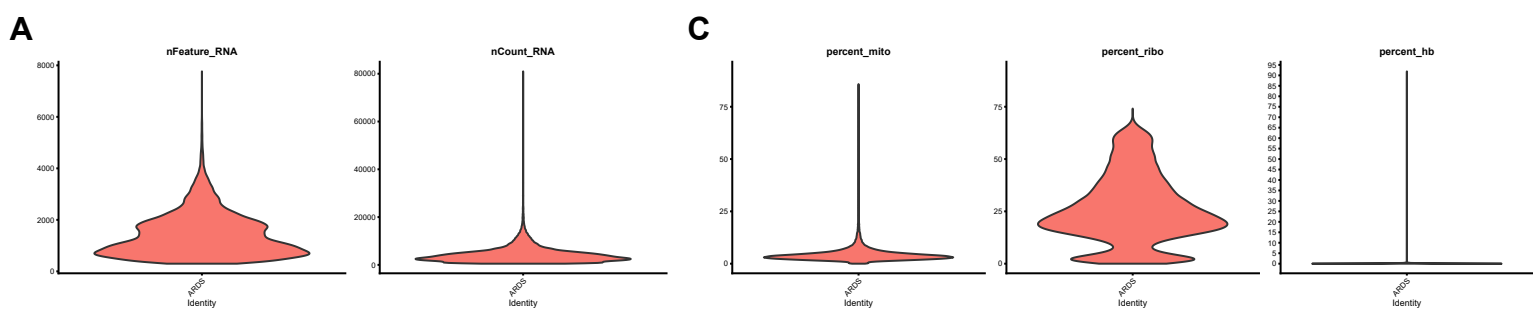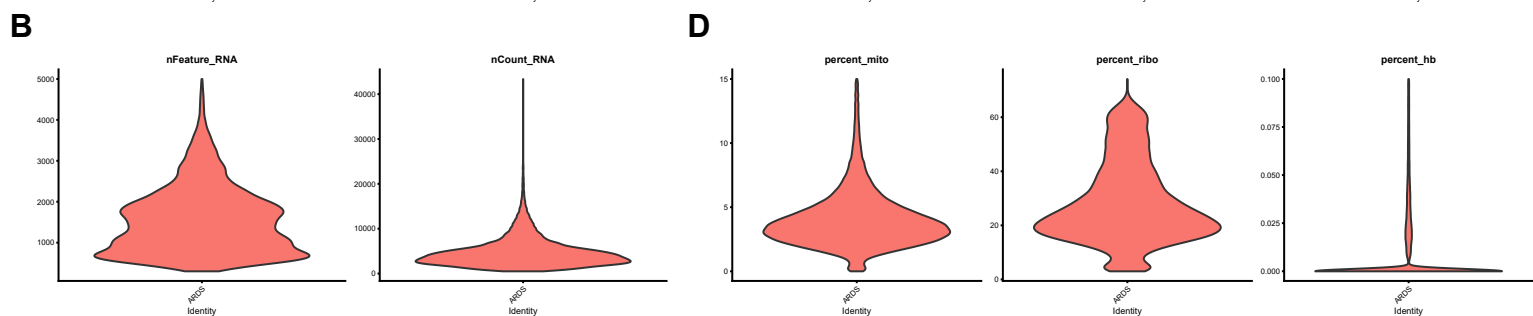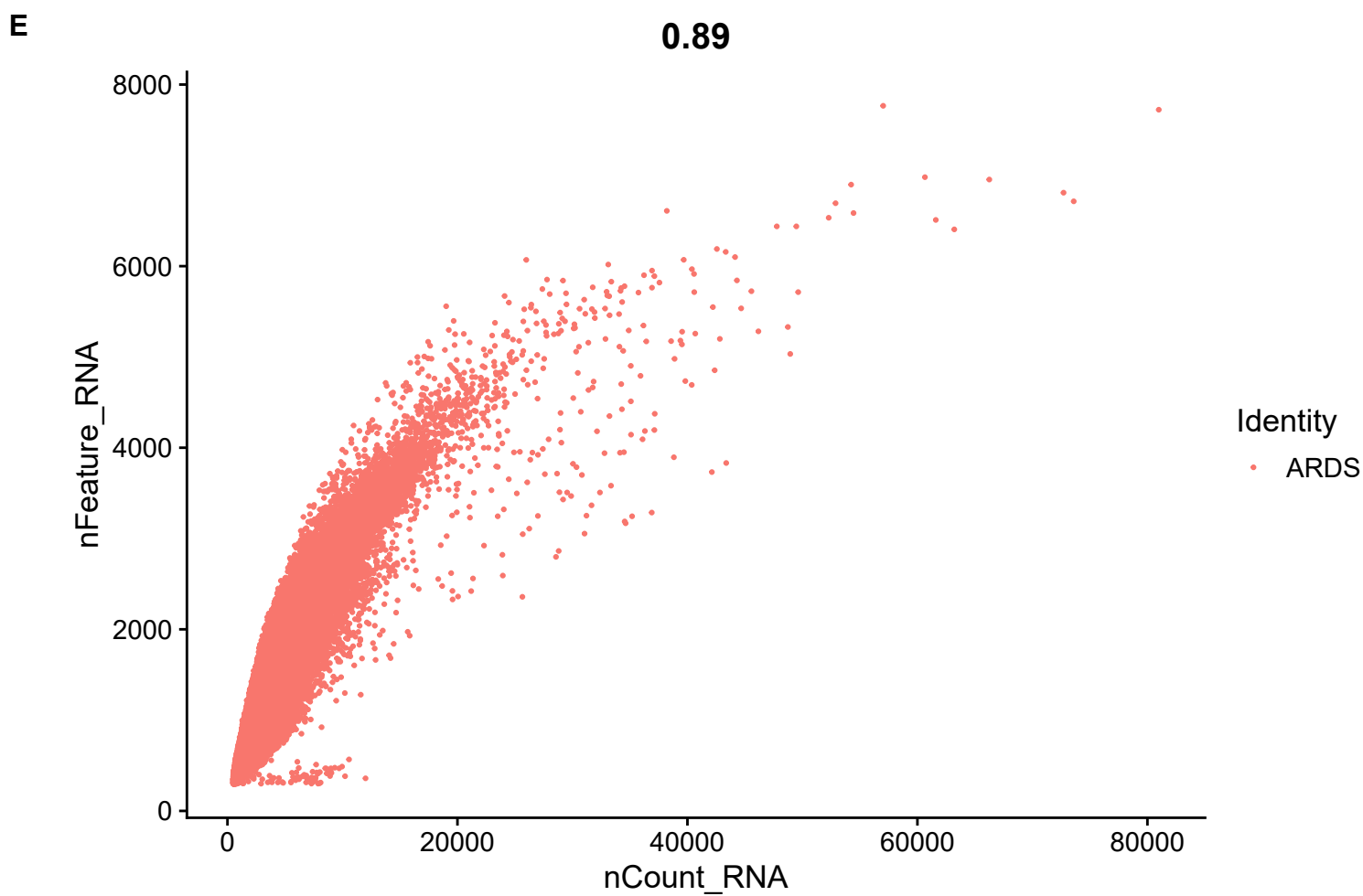

Supplement: Supplementary Figure 1 — Single-cell data preprocessing and rigorous quality control. scRNA-seq data were integrated from GEO (GSE151263 and GSE242127) and bulk data from GSE66890. The 336 lactylation genes were curated from high-impact literature (PMIDs: 37242427, 35761067, 36092712). Analysis used R 4.2.2 and the Seurat package with Harmony batch-effect mitigation. Quality control (QC) criteria: the number of genes detected per cell (nFeature-RNA) is less than 5000, the mitochondrial gene expression ratio (percent_ito) is less than 15%, the ribosomal gene expression ratio (percent_ribo) is greater than 3%, and the hemoglobin gene expression ratio (percent_rb) is less than 0.1%. 46,192 cells were retained. (A, B) Violin plots of nFeature_RNA and nCount_RNAn pre- and post-QC. (C, D) Distribution of mitochondrial, ribosomal, and hemoglobin genes pre- and post-QC. (E) Correlation between nFeature_RNA and nCount_RNA, indicating high data fidelity. Cell types were annotated using SingleR and manual curation (Resolution 0.8). [file Image1.pdf]

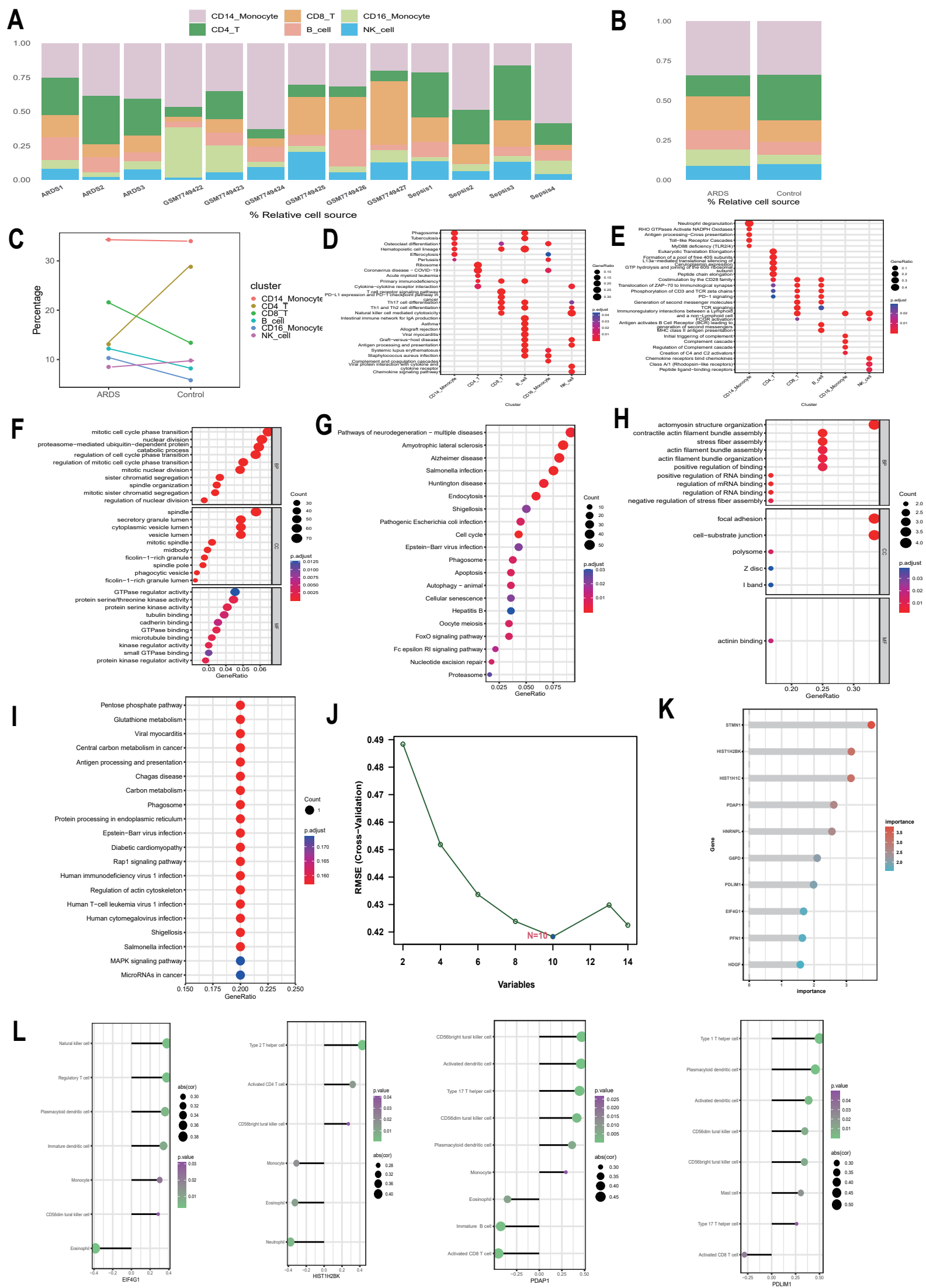

Supplement: Supplementary Figure 2 — Systematic bioinformatics pipeline for core gene prioritization. (A) Cellular composition per sample. (B, C) Proportional distribution and trends of cell types across groups. (D, E) KEGG and Reactome pathway enrichment of top 100 cluster markers. (F) GO (BP, CC, MF) and KEGG enrichment of differentially expressed genes (DEGs). (G) Summary of single-cell differential analysis (4,007 up-regulated, 408 down-regulated DEGs). (H, I) Functional GO and KEGG enrichment of the intersected DEGs. (J, K) Top 10 genes identified via SVM (J) and Random Forest importance ranking (K). (L) Correlation map between the 4 core hubs and significantly infiltrating immune cells (ssGSEA; dot size: coefficient; color: pp-value). [file Image2.pdf]

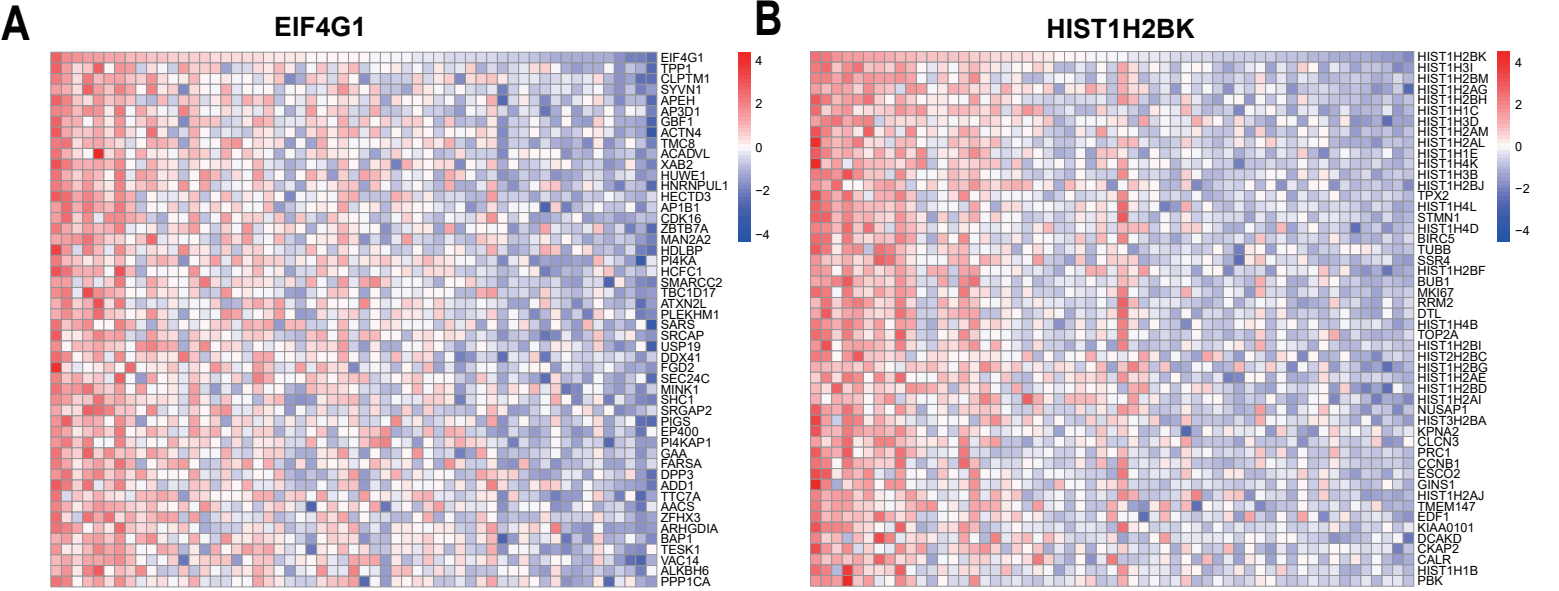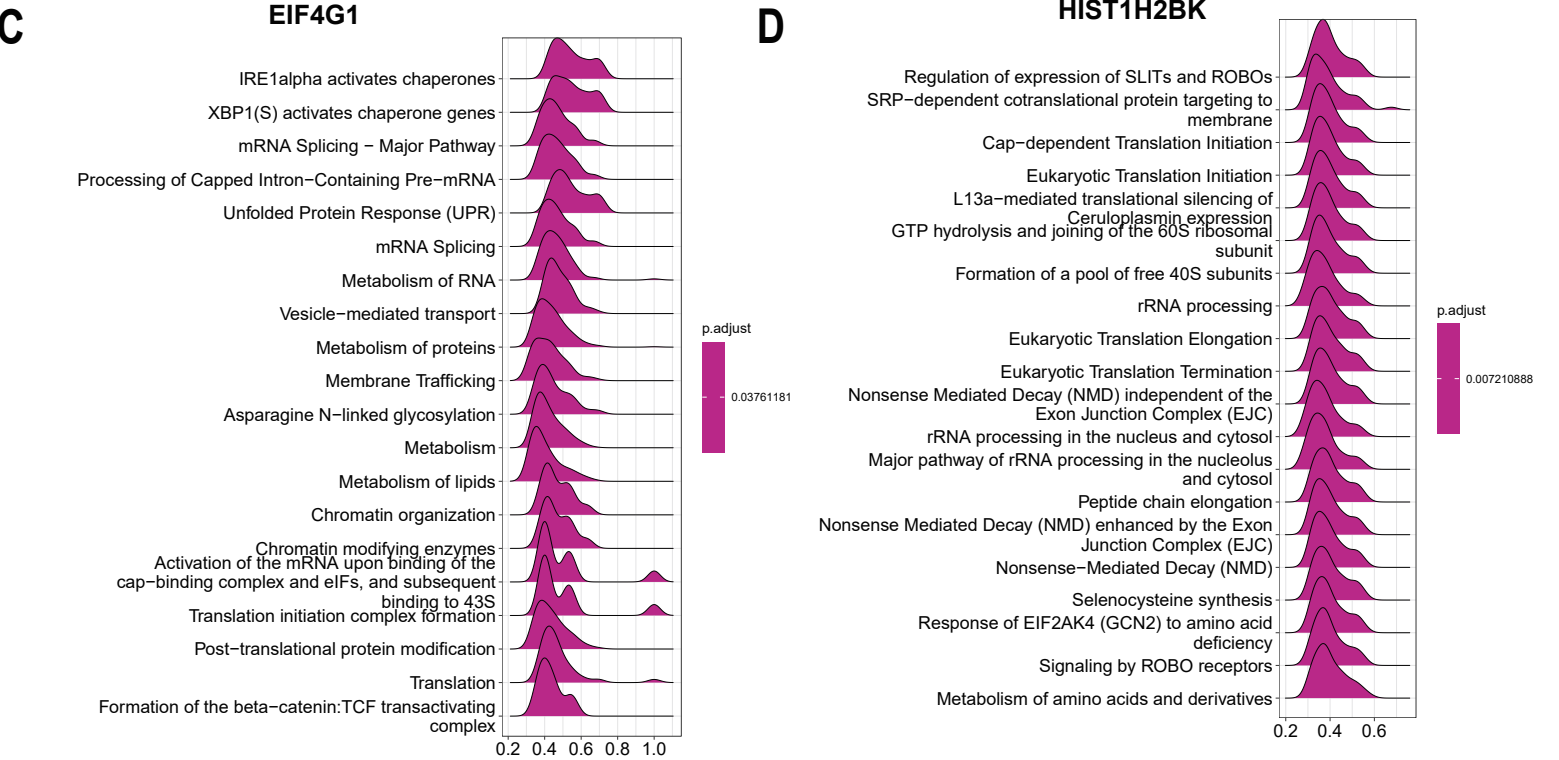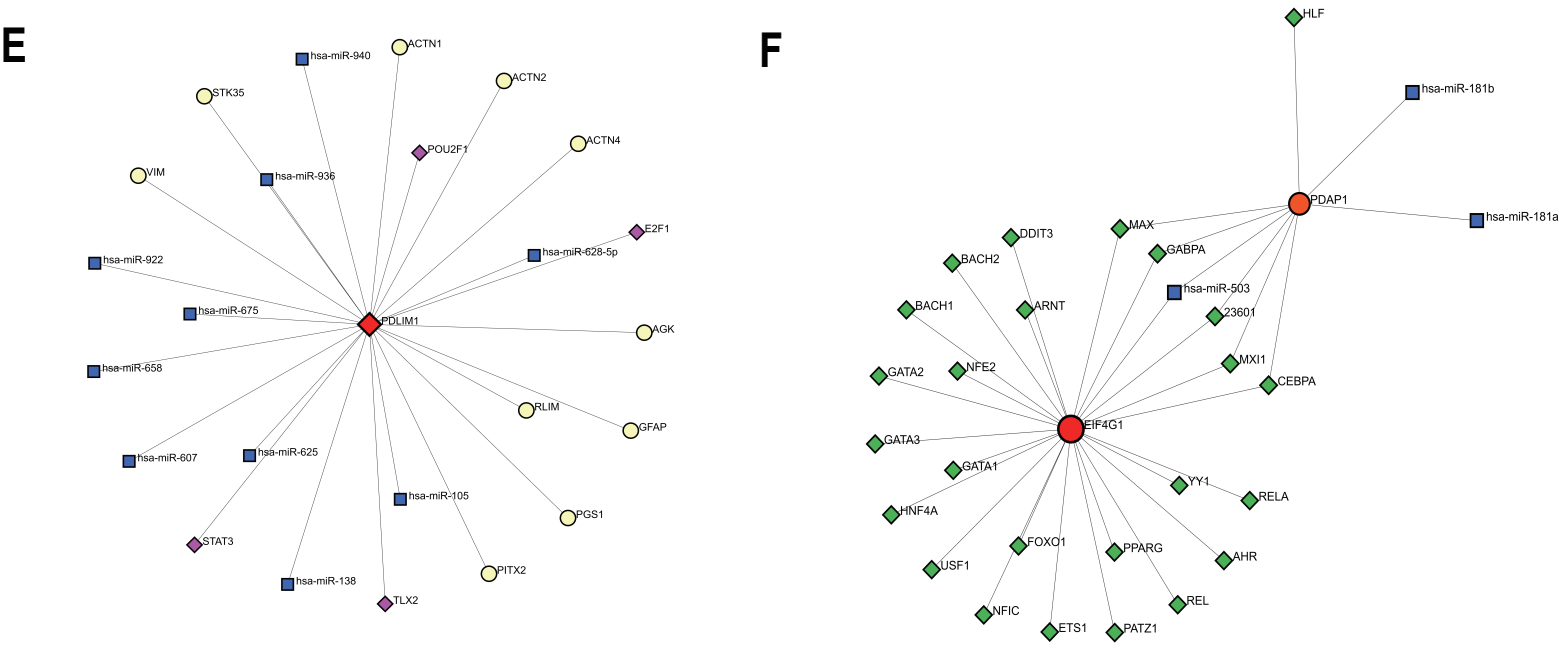

Supplement: Supplementary Figure 3 — Upstream regulatory networks and functional enrichment of EIF4G1 and HIST1H2BK. (A, B) Genome-wide correlation heatmaps for EIF4G1 and HIST1H2BK, highlighting the top 50 co-expressed genes. (C, D) Single-gene Reactome GSEA (Top 20 pathways) for EIF4G1 and HIST1H2BK; enrichment scores indicate respective pathway correlations. (E, F) Upstream miRNA-TF regulatory networks for the core hubs predicted via NetworkAnalyst (displaying significant nodes). [file Image3.pdf]
